# Socioeconomic importance of the semi-feral goat population for smallholders on the island of Socotra (Yemen)

**DOI:** 10.1007/s11250-025-04290-1

**Published:** 2025-02-10

**Authors:** Lucie Maděrová, Jan Šipoš, Petr Maděra, Josef Suchomel

**Affiliations:** 1 Department of Zoology, Fisheries, Hydrobiology and Apiculture, Mendel University, Zemedelska, Brno, 61300 Czech Republic; 2 Department of Forest Botany, Dendrology and Geobiocoenology, Mendel University, Zemedelska, Brno, 61300 Czech Republic

**Keywords:** Goat, Milk production, Socotra archipelago, Reproduction, Management practices

## Abstract

We examined the socioeconomic importance of goat farming on the island of Socotra. The study included 154 participants from various areas. These data were collected via a questionnaire and statistically analyzed using the Bayesian approach and Kruskal–Wallis test to offer insights into the subject matter. The collected data included important quantitative parameters, such as feeding, watering, herd composition, milk production, and mortality, as well as subjective parameters, including the breeders’ reasons for keeping goats. Our study revealed that the main reason for keeping goats on Socotra Island is livelihood purposes (60%), followed by cultural reasons (22%), whereas 18% of respondents keep goats as a companions. In terms of herd composition, generally a Socotri family keeps an average of 107 individuals per herd, of which 59% are adult females, 32% are young under six months, and 9% are adult males. Reproduction patterns indicate that the goats breed twice a year, primarily in May and October. For milk production, goats on Socotra produce an average of 368 ml/day on an annual basis, and the highest production is observed in the mountains. The study also revealed that goats are primarily slaughtered for social reasons, such as family attendance and weddings (55%). According to the respondents, the main cause of mortality in goats was disease (40%). The results of this study have implications for understanding the management of goat farming on the island and conserving the island’s biodiversity.

## Introduction

The Socotra Archipelago, located approximately 380 km south of Ras Fartak on the Gulf of Aden and approximately 230 km east of Cape Guardafui in Somalia, is renowned for its endemic flora and fauna (Van Damme 2009).

The ancient continental origin places the separation of the island from the mainland ca. 20–17 million years ago (Culek 2013); together with the climatic conditions and diverse topography, this has resulted in remarkable endemic biodiversity (Van Damme 2009). Many endemic plant species (320, i.e., 37% of the specific endemism rate) and animals motivated its designation as a United Nations Educational, Scientific and Cultural Organization (UNESCO) World Natural Heritage site in 2008. The site is also home to globally important populations of birds, including several endangered species (UNESCO 2023). Notably, terrestrial biodiversity faces significant threats due to domesticated animals, particularly goats. This is related to the changes in traditional patterns in the management of goat populations (Van Damme and Banfield 2011), which are allowed to roam freely across the island despite each animal having an individual owner (Miller and Morris 2004). Accordingly, the goat population in Socotra is in a semiferal state, posing a significant challenge to the delicate balance of the ecosystem. Compared with the average number of goats in other climatically similar regions, the fivefold greater number of goats kept by one family on Socotra leads to fundamental overgrazing of the island’s vegetation and prevents the natural regeneration of tree species, as mentioned by several studies (Hamdiah et al. 2022; Lvončík et al. 2020; Maděra and Van Damme 2022; Rezende et al. 2022; Maděra et al. 2019a, b; Habrová and Pavliš 2017).

Livestock on Socotra include, in order of abundance, goats, sheep, cattle, camels, and donkeys (Scholte et al. 2007). The domestic goat (*Capra hircus*) probably appeared on Socotra with the arrival of the first humans, at least from 2,000 years ago (Scholte et al. 2007) up to 6,000 years ago (Černý et al. 2009). Before the mid-twentieth century, nomadic pastoralism was the prevailing mode of animal husbandry. The practice involved a system of rotating pastures, whereby people would move their animals to areas with sufficient rainfall and grassland. However, following World War II, the settlement patterns of many pastoral communities became more sedentary, leading to a shift away from nomadic pastoralism and toward a system of free grazing. This change was largely driven by a significant increase in the number of goats raised, which made it difficult to effectively rotate pastures (Scholte et al. 2007). Similar transitions away from nomadic pastoralism have been documented in other arid regions in the world, including Sudan (Vetaas 1993).

The people of Socotra are strongly bound to their livestock. This strong attachment to livestock has a historical and cultural dimension that goes beyond their mere perception of the animals as a source of food. Goat rearing is at the heart of Socotra’s uniqueness. As written by Scholte et al. (2007), if you exclude goats, you lose the essence of what makes Socotra special: the combination of endemic fauna and flora - and goats. Nevertheless, livestock is still increasingly held as financial assets (along with income from other sources such as fisheries) (Scholte et al. 2007). In additon, goats are also subject to a system of taxes (Elie 2008).

Goat grazing depends on the season and is also limited by the monsoon, during which the economy comes to a standstill, according to Elie (2008). Goats are numerous and move freely in all ecosystems, from the mountain tops to the capital (personal observation). While in 1966, the number of goats was estimated at approximately 19,000 (Brown 1966), it was already 220,000 in 1999 (Scholte et al. 2007). According to the last census, there are 480,000 goats on Socotra (Maděra and Van Damme 2022), corresponding to an average of 1.33 goats per hectare; this number does not include sheep, cattle or camels (Maděra and Van Damme 2022). Goat populations have historically fluctuated dramatically, mainly as a function of rainfall. Regarding mortality, as argued, local people in Socotra have also described the drought years when the livestock populations plummet. Severe winter floods after summer rainfall are even more disastrous than these droughts are, as weakened cattle die from the cold (Miller and Morris 2004). However, the introduction of new management practices such as water provision, veterinary care, supplementary fodder, and transport of animals by trucks to other areas has increased their survival during droughts (Scholte et al. 2007).

Only a relatively small body of literature is concerned with the goats of Socotra. Our work aimed to delve deeper into this topic and explore it further. Specifically, areas of interest include goat farming, herd composition, reproduction, milk production, supplementary feeding and watering, and slaughter and nonslaughter mortality. A comparative analysis of certain parameters was conducted across the island’s distinct seasons (dry and rainy), as well as various areas (highlands and lowlands).

## Materials and methods

### Data collection

A survey was conducted through personal interviews with local people on Socotra. The average age of the interviewees was 30 years (range: 14–65), and 100% were male. Households comprised an average of 9 members (range: 0–25). The mean household income was $108, with 63% earning $0–80, 16% earning $85–100, and 19% earning $120–1000. A questionnaire was designed to obtain information from respondents on the demography of goats (see Table B presented in the appendices). There was also an interpreter who could speak English, Arabic, and Socotri and explain the questions. The questions were posed in the Socotri and Arabic languages. A sample of 154 households was selected to ensure adequate representation of the diverse ecological zones of Socotra. The selected sample groups were designed to ensure that residents from different geographical regions, namely, mountains (11% of respondents), lowlands (50% of respondents), and highlands (39% of respondents), were equally represented. The distribution of respondents correlated with the quantitative representation of goats by geographic location. Specifically, a large proportion of the island’s surface area (48%) is below 400 m above sea level and is classified as a lowland. The lower highlands, ranging between elevations of 400 and 700 m, account for 43.5% of the island’s surface area. Finally, mountainous areas more than 1,200 m above sea level constitute a mere 0.3% of the island’s surface area (Brown and Mies 2012). The questionnaires were distributed in two periods: the rainy season (October 2021) and in the dry season (March 2022). Table 4 (presented in the appendices) serves as a means to provide supplementary information about the surveyed individuals. We used the topographical names in accordance with Bezděk et al. (2012).

### Description of the study site

This study was carried out on Socotra Island (12.19°–12.42°N latitude and 53.18°–54.32°E longitude). The island of Socotra belongs to the Republic of Yemen and is located in the northwestern part of the Indian Ocean. Socotra occupies 3,600 km^2^ (Van Damme and Banfield 2011), with an estimated population at 80,000, with seasonal variations. However, this number is uncertain because there is no census in Socotra (Maděra and Van Damme 2022). The East African-Indian monsoon system strongly influences the climate of Socotra, with a biannual migrating intertropical convergence zone between the northern hemisphere winter position and the southern Indian Ocean summer position (Scholte and De Geest 2010). The summer monsoon accurs from May to September, and the winter monsoon lasts from October to January (Scholte and De Geest 2010; Kalivodová et al. 2020).

### Statistical analysis

We adopted a Bayesian approach to investigate the influence of various environmental characteristics on key socioeconomic aspects of the Socotri pastoral community, as follows: milk production (*ml*), herd composition of goats ($N_{composition}$), slaughtered goat age ($N_{sloatherage}$) and supplementary goat watering ($N_{watering}$) and feeding (1/0). We used the “|brm|” function, which is part of the widely used “|brms|” package in R, to fit Bayesian regression models with the crossed random intercept effect set as locality (Bürkner 2017). In the model, we set noninformative priors to model the uncertainty in the model parameters. The specification of the response distribution depended on the type of response variable being analyzed.

For count data, such as the number of activities occurring within a fixed time period, a zero-initiated Poisson (e.g., slaughtered goat age, goat watering, or milk production) or a Poisson distribution was utilized (e.g., number of adult goats or goat offspring and pregnant females). Finally, a Bernoulli distribution was used when dealing with binary outcomes (e.g., supplementary feeding).

We specified four chains in the model, each with 2,000 iterations, using Markov chain Monte Carlo (MCMC) methods to achieve convergence and good mixing across multiple chains. Analyzing convergence diagnostics is crucial in assessing the credibility of model outcomes, as highlighted by Gelman and Rubin (1992) and Brooks and Roberts (1998). A scale reduction factor (SRF) greater than 1.2 or less than 1.0 suggested that the parameters may not have reached convergence within that chain. The values of the SRF, also known as the Gelman–Rubin statistic, were below the 1.2 threshold for our models, indicating convergence. In the Bayesian model, we specified the random seed as 123 to ensure the reproducibility of the results across different runs.

We also applied the Kruskal-Wallis test to compare multiple groups (e.g., reason for keeping goats, diet composition, slaughter reasons, reproduction and nonslaughter mortality), where assumptions of normality and homogeneity of variances were violated. All constructed tests were carried out in the R programming environment.

## Results and discussion

### Reason for keeping goats

**Fig. 1:**
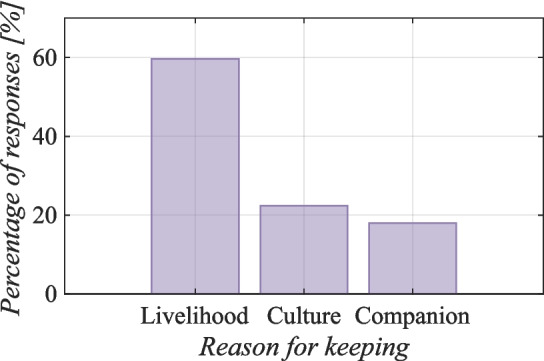
The main reasons for keeping goats on Socotra Island according to 154 respondents (Kruskal–Wallis rank sum test, value by meaning Kruskal–Wallis $\chi ^2 =141.28$, $df = 2$, $p =< 2.2 \cdot 10^{-16}$)

Figure 1 illustrates that the primary reason for breeding goats is for their livelihood (60%) ($p<$ 0.001). Goats provide a means of sustenance for Socotri herders, as they are used for meat and other products. Milk can be consumed directly or with rice (as sour milk). Goat meat is primarily used for individual sustenance and social events such as weddings and guests or sold on the market. Socotri people generally consume limited quantities of goat meat for personal purposes. The fur is utilized for commercial purposes, such as to produce clothing and rugs, sold at markets in some regions of Socotra. Goatskin can be used to store various liquids and foods to extend their shelf-life. Leather is also used to prepare knife sheaths, feeding bottles, floor mats, mattresses, and waterproof roofing materials (Miller and Morris 2004).

Furthermore, goats are a significant element of the culture (22%) ($p<$ 0.001). They hold importance in Islamic mythology and symbolize wealth. They are also used for ritual slaughter or as a dowry for a bride’s wedding. Finally, some Socotri people answered that they kept goats as pets and cherished them as they would their own children (18%) (18%) ($p<$ 0.001).

**Table 1:** The purpose of keeping goats on Socotra compared with other places

Place	The Purpose of Keeping Goats	Source
Socotra (Yemen)	Milk production, sour milk, meat, funeral, wedding, feast, sale, ritual slaughter, symbol of wealth, companionship, fur for carpets/handicrafts/belts/headband, goat skins for storing dates, aloe, milk, buttermilk and water/floor mats/mattresses/waterproof roofing material/knife sheath/feeding bottle.	This study, (Miller and Morris 2004)
Mgwalana (South Africa)	Traditional ceremonies, sales, domestic meat consumption.	Mahanjana and Cronje (2000)
Niger (West Africa)	Meat, skin, and milk production.	Agossou et al. (2017)
Nigeria (West Africa)	Meat production, skin production, milk production, wedding, funeral, gift.	Chaniago (1993); Agossou et al. (2017)
Alaba (Southern Ethiopia)	Domestic milk consumption.	Ketema (2007)
Israel (Middle East)	Milk production, wool, hair (for carpets and tents), meat production, butter, ghee, yogurt, dried curd, sale.	Degen et al. (2000)
Buyende District (Uganda)	Insurance for hard times.	Nampanzira et al. (2015)
Indonesia (Southeast Asia)	Meat production.	Bradford (1993)
Zimbabwe (Southern Africa)	Meat production, manure, income, symbol of wealth, skins, cultural purposes, ceremonies, milk production.	Mhlanga et al. (2018)
Ethiopia (Amhara region)	Cash income, meat, manure for fertilizer, milk production, cheese, butter, yogurt.	Abegaz (2014); Alemu (2015); Taye et al. (2024)

The global practice of breeding goats is driven by numerous factors. The reasons for breeding goats worldwide are similar to those for breeding goats on Socotra, as shown in Table 1.

**Fig. 2:**
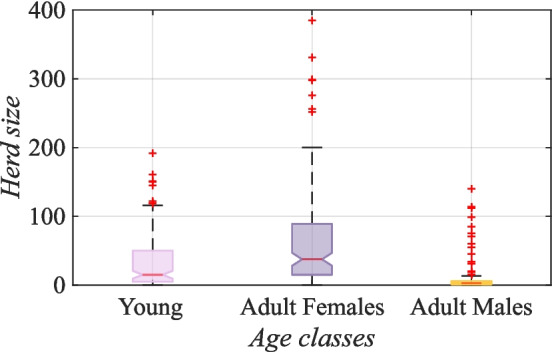
Herd composition of goats on Socotra (Bayesian statistics, l-95% CI= −1.93, u-95% CI= −1.82 for adult males; l-95% CI= −0.63, u-95% CI = −0.56 for young). The box represents the upper (75^th^ percentile) and lower (25^th^ percentile) quartiles, with the red line indicating the medial value and two whiskers representing the maximum and minimum values of herd composition of goats without outliers corresponding to approximately to $\pm 2.7\sigma$, with the notch representing the 95% confidence interval of the median

### Herd composition

Figure 2 shows that the average herd size of the goats on the island was 107 individuals per herd, which is a relatively large number. In an average herd, 59% were adult females, 32% were young under six months, and 9% were adult males. Notably every respondent from the sample group kept goats (100%).

The larger the family is, the more goats are kept (Bayesian statistics, l-95% CI= 0.070, u-95% CI = 0.244 for a number of family members). Notably, in Mgwalana, only 86% of households keep goats, and the average herd size is only 16 individuals (Mahanjana and Cronje 2000). In Kano (Nigeria), the average herd size is 15.5 goats (Akpa et al. 2010); in Al-Jabal-al-Akhdar (Northern Oman), it is 25 goats (Dickhoefer 2006); in Benin (West Africa), it is 10 goats (Koura et al. 2024); and in Buyende District, it is 13 goats (Nampanzira et al. 2015). The migratory Raika people (India) keep 24 goats (Geerlings 2001), and in Kiang West, Niamina East and Nianjia (Gambia), there are 15 goats (Ejlertsen et al. 2012), and in Pakistan (Gilgit Baltistan regions), on average, there are 15 goats per household (Abdullah et al. 2024). This difference can be explained by the fact that goats on Socotra are the primary source of livelihood for herders’ families (see Fig. 1). In addition, as described by Cheung and DeVantier (2006), the Socotri and their goats have a strong bond that extends beyond the herders’ mere perception of the animals as a food source. This was confirmed by our study, as 18% ($p<$ 0.001) of the respondents reported that they regarded goats as their pets. Moreover, a study in Mgwalana reported that local people breed goats mainly for ritual slaughter, do not consume much milk from goats (only 30%), and do not eat much goat meat. The reason for the discrimination against goat meat was attributed to its taste and smell by 76% of the respondents, which is the opposite of the opinion in Socotra. In contrast, in Jabal-al-Akhdar, the primary purpose of raising goats is meat production (Dickhoefer 2006).

Notably, the territory in which a goat grazes is often quite large, and goats are often transported to different areas for browsing at different times. People live in tribes and divide their lands; however, in times of drought, tribes may agree among themselves and move their animals to areas with better grazing. However, many land disputes have occurred in the past when people put goats where they were not allowed, and the goats have caused problems (Van Damme and Banfield 2011). Therefore, agreements between farmers are significant. When these agreements are worked out, they can optimize the number of herds, whereas in other nations, the number of goats is limited by the size of the land or neighboring farmland.

On Socotra, the ratio of adults females and males is 87% to 13%. According to a study by Akpa et al. (2010), in Kano, the ratio of female to male goats is 80% to 20%. In northern Mexico, the ratio is 3% to 97% for adult females (Mellado et al. 1996). A study from Jordan (Mafraq Governorate) reported a 94 to 6% ratio of adult females to adult males (Al-Barakeh et al. 2024). In Ethiopia (Arado), the adult male-to-female ratio is 16% to 84% (Mekonnen et al. 2024). This is not surprising because adult female goats can be impregnated, and therefore, herders can use their milk and raise their young for meat and barter. There are few adult males because they are only used for breeding. In Tanggamus Regency (Indonesia), the ratio of male to female goats was reported to be 50% to 50% in 2018, according to a study by Sulastri et al. (2019).

In our study, other results show that a significant portion of goat farming is concentrated in mountainous areas (Bayesian statistics, l-95% CI= −0.35, u-95% CI= 0.13 for lowlands; l-95% = 0.76, CI u-95% = 0.95 for mountains; l-95% CI= −3.17,u-95% CI= −1.41 for cities). The average number of goats per family in the high mountains (above 1,200 meters above sea level) is 185 (n=185), which is significantly greater than in other areas, such as highlands (n=79), lowlands (n=67), and cities (n=8). This phenomenon can be interpreted as a consequence of mountain dwellers’ dependence on goat products. Due to their geographic distance from towns, local people depend mainly on local resources, such as goats and rice, for their food and economic needs.

In the present study, this statement was confirmed by further analysis, which revealed that the number of young born in the mountainous areas was the highest, which may be linked to the beneficial environmental conditions in the mountains (rich pastures, green all year) and perhaps a preference for slaughtering goats at a younger age in these areas. On average, for one family, 37 young are born per year in the mountains (n=37) (Bayesian statistics, l-95% CI= 0.78,u-95% CI= 1.13 for mountains) compared with lowlands (n=17), highlands (n=14) and cities (n=5). The average age of a goat to be slaughtered in the mountains is three months (n=3), which is younger than that in other terrain types, such as lowlands (n=5), highlands (n=9), and cities (n=7). Overall, these statistics suggest that goat farming in the mountainous areas is essential to the livelihoods of local people.

**Fig. 3:**
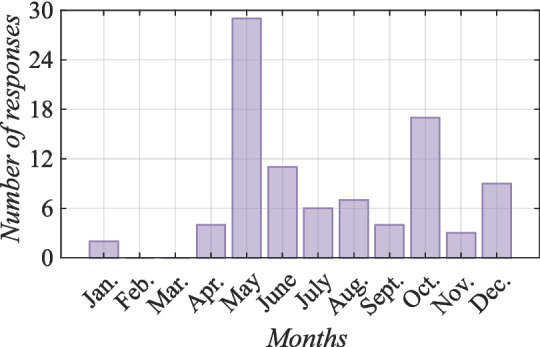
Annual distribution of reproduction periods on Socotra (Kruskal–Wallis rank sum test, value by reason Kruskal–Wallis $\chi ^2 = 98.70$, $df = 11$, $p = 3.24 \cdot 10^{-16}$)

### Reproduction

According to the information provided, Fig. 3 illustrates the reproduction period of adult females on Socotra. It was observed that goats reproduce twice a year, with peak occurrences in May and October ($p<$ 0.001). Additionally, although less frequently, instances have been noted in April, June, July, August, September, December, and (least frequently) January ($p<$ 0.001). This distinctive pattern can be attributed to the inherent seasonality of reproduction in goats, as well as the strategic breeding practices employed by local breeders, who often opt for controlled mating during the rainy season.

Reproductive seasonality is common in goats living at temperate and subtropical latitudes. Adult females are normally receptive from early autumn to late winter (Chemineau et al. 1992; Gallego-Calvo et al. 2014). This is also consistent with the finding that goats are let in quite often in October and December. The female becomes pregnant during the rainy season, which runs from May to September (Kalivodová et al. 2020) and from October to January (Scholte and De Geest 2010), meaning that there is enough food to cover the energy needs for fetal development. The same reproductive strategy is used, for example, in Mexico (Mellado et al. 1996). Thus, in this zone, mating at the beginning of the rainy season (June) ensures acceptable reproductive performance. However, the milk of adult females and the young growth rate and survival are compromised because lactation and offspring development occur when the forage growth and nutrient content of the diet of goats are at a minimum.

**Fig. 4:**
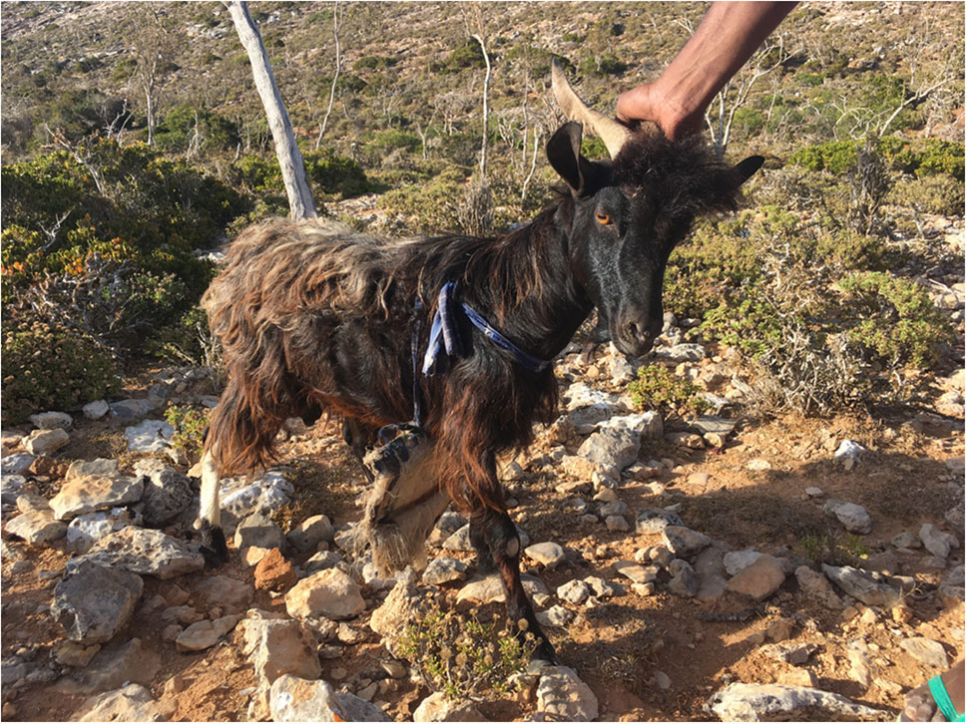
Socotri adult male with mating protection. Photo by Lucie Maděrová, Socotra 2021

On Socotra, there are no structured breeding systems or appropriate infrastructure, such as paddocks; therefore, adult females and males run together throughout the year. There is reproduction planning to synchronize kiddings with pasture, which shows indigenous knowledge of how to conduct reproduction. This is why the locals put a protection shield (Fig. 4) to prevent mating. It is made of various materials, such as bark from a tree, specifically *Adenium obesum* (Miller and Morris 2004), straw or a car tyre fender. During the mating season, breeders take the protection shield off of their adult males. This is an interesting and practical method that could inspire better breeding management, for example, for breeders in Mgwalana, where adult females and males are never separated, and there is no fixed age or time for castration of adult males. When respondents were asked to provide reasons for not practicing controlled breeding, 90% attributed this to the communal land tenure system (i.e., the lack of camps and fencing) and to a lack of labor or time (Mahanjana and Cronje 2000).

### Supplementary feeding and watering

In the context of Socotra, the practice of free-ranging goats has been observed to be accompanied by systematic support of the nutrition of these animals. According to local knowledge, goats are supplementarily fed once a day during the dry season; however, during the rainy season, the frequency of supplementary feed is reduced to once every two days (Bayesian statistics, l-95% CI= 0.60, u-95% CI = 1.08 for the rainy season). According to Mataveia et al. (2019), goats were also primarily provided supplements during the dry season in the Namaacha and Moamba districts (southern Mozambique) when feeding resources were scarce. This variation in feeding practices is largely a response to seasonal changes in grazing availability. During the rainy season, for example, green cover is more abundant, and goats have access to richer food resources, which reduces the need for frequent supplementary feeding. Conversely, in the dry season, when food availability is limited, the frequency of supplementary feeding increases.

**Fig. 5:**
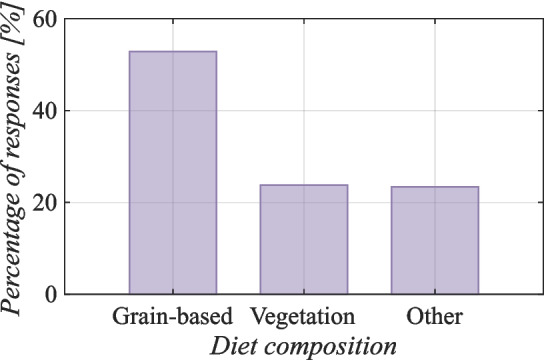
Composition of the goat diet on Socotra (Kruskal–Wallis rank sum test, value by reason Kruskal–Wallis $$\chi ^2 = 27.08$$, $$df = $$2, $$p = 1.32 \cdot 10^{-6}$$)

Figure 5 shows the main sources of supplementary food for the goats. The results of this study’s survey suggested that the goats are fed various vegetation, including leaves and bark from trees and various plants. They are also fed grain-based foods, including finger millet and wheat. Other food includes human dietary residues (mainly rice) and commercially available compound feeds or dates. Some respondents also reported giving goats paper pages torn from books as food, as providing complementary sources of fiber is believed to be beneficial for animal health. Additionally, dried sardines are sometimes added to the goats’ diet to improve the nutritional value of the food with vitamins and minerals ($p<$ 0.001). In other tropical areas, such as Al-Jabal-al-Akhdar, goats are fed rice, fish, bread, or wheat, as reported by Dickhoefer (2006). Similarly, in Buyende District (Uganda), local farmers reported that goats are fed various forages, predominantly from natural pastures. Other food sources in these areas include crop residues, such as sweet potatoes, maize, millet or sorghum, rice straw, and byproducts of food crops. In addition, some farmers use compound feed (Nampanzira et al. 2015).

**Fig. 6:**
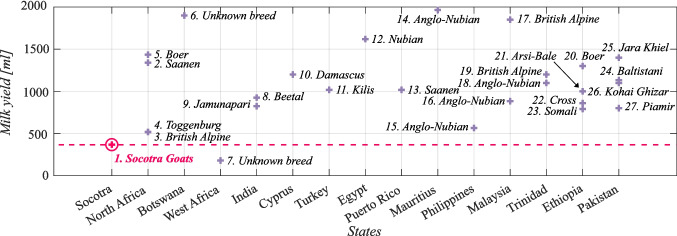
Comparison of milk yields based on country and goat breed ( The red cross represents the average volume of milk production in goats of the Socotra region. A comparison of this milk yield with that of other goat breeds from various countries is illustrated by a dashed line)

**Table 2:** Goats milk yield in tropical countries

No.	Place	Breed	Source	No.	Place	Breed	Source
1	Socotra	Local Socotri breed	This study	15	Philippines	Anglo-Nubian	Devendra and Burns (1983)
2	North Africa	Saanen	Norris et al. (2011)	16	Malaysia	Anglo-Nubian	Devendra and Burns (1983)
3	North Africa	British Alpine	Norris et al. (2011)	17	Malaysia	British Alpine	Devendra and Burns (1983)
4	North Africa	Toggenburg	Norris et al. (2011)	18	Trinidad	Anglo-Nubian	Devendra and Burns (1983)
5	North Africa	Boer	Sahni and Chawla (1982)	19	Trinidad	British Alpine	Devendra and Burns (1983)
6	Botswana	Unknown breed	Dipheko et al. (2016)	20	Ethiopia	Boer	Mestawet et al. (2012)
7	West Africa	Unknown breed	Jaitner et al. (2006)	21	Ethiopia	Arsi-Bale	Mestawet et al. (2012)
8	India	Beetal	Prakesh and Khanna (1972)	22	Ethiopia	Cross	Mestawet et al. (2012)
9	India	Jamunapari	Prakesh and Khanna (1972)	23	Ethiopia	Somali	Mestawet et al. (2012)
10	Cyprus	Damascus	Constantinou (1981)	24	Pakistan	Baltistani	Abbas (2003)
11	Turkey	Kilis	Yarkin and Eker (1961))	25	Pakistan	Jara Khiel	Abbas (2003)
12	Egypt	Nubian	Wilson et al. (1991)	26	Pakistan	Kohai Ghizar	Abbas (2003)
13	Puerto-Rico	Saasen	Devendra and Burns (1983)	27	Pakistan	Piamir	Abbas (2003)
14	Mauritius	Anglo-Nubian	Devendra and Burns (1983)				

With regard to watering their animals, goats on Socotra drink from both natural sources, such as wadis, pools, and streams, and artificial sources, such as rainwater tanks (kareefs), dug wells, and artificial pools (leems) , when left free. Similar practices have been documented in Alaba, where goats mainly drink from rivers, artificial ponds, troughs, and harvested water (Ketema 2007). While goats are a species of animals that are extremely resilient to dry environments and have an extraordinary ability to cope with dehydration (Ronda-Borzone et al. 2022), in times of drought, these water sources often dry out, leading people to actively water their herds. The frequency of this watering again varies depending on the season. During the dry season, watering is performed once a day, whereas during the rainy season, it is performe once every six days (Bayesian statistics, l-95% CI= 1.66, u-95% CI= 2.04 for the rainy season).

### Milk production

The amount of milk produced varies significantly among the breeds of goats (see Fig. 6 and Table 2). The Socotra yield, on average, is 368 ml/day annually, considering that the lactation period for these goats lasts 150 days.

**Table 3:** Comparison of milk production in dry and rainy seasons.

Place	Rainy (ml/day)	Dry (ml/day)	Source
Socotra (Yemen)	1,195	916	This study
Sesfontein (Namibia)	875	263	Degen (2007)
Eastern Ethiopia	370	220	Baars (2000)
Adamawa (Nigeria)	460	280	Midau et al. (2010)

Tropical regions have alternating rainy and dry seasons. Therefore, it is necessary to compare goat milk yields during these periods. In Africa, similar seasonal differences are observed, as shown in Table 3. There was a statistically significant (Bayesian statistics, l-95% CI= 0.43, u-95% CI= 0.44 for the rainy season) difference in milk production between the dry and rainy seasons in all investigated areas, as shown in Table 3. In Socotra, goats produce an average of 1,195 ml/day in the rainy season and 916 ml/day in the dry season. This confirms the findings of Gulelat (2002), who reported that milk production by goats depends on forage and water availability and, consequently, is lower during the dry season and higher during the rainy season.

**Fig. 7:**
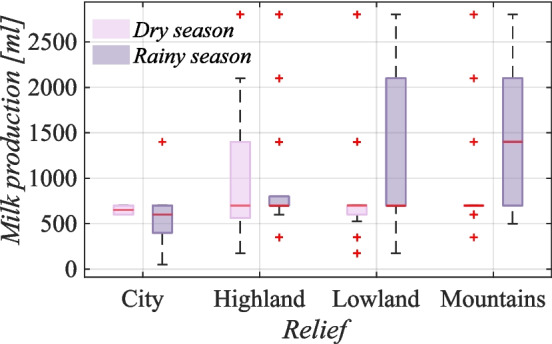
Influence of relief and seasonality of milk production on Socotra (Bayesian statistics, l-95% CI = 0.38, u-95% CI = 0.42 for lowlands; l-95% CI= 0.38, u-95% CI= 0.43 for mountains; l-95% CI= −0.34, u-95% CI = −0.21 for cities). The box represents the upper (75^th^ percentile) and lower (25^th^ percentile) quartiles, with a red line indicating the median value and two whiskers representing the maximum and minimum values of herd composition of goats without outliers corresponding to approximately $\pm 2.7\sigma$

If we investigate the study of milk yields at different altitudes, we can extract important information about the impact of the dry and rainy seasons on milk production. The results depicted in Fig. 7 reveal that in the lowlands, goats produce more milk in the rainy season, at a rate of 1,280 ml/day, than at only 825 ml/day.

Similarly, in the mountains, the milk yield is greater in the rainy season at 1,535 ml/day compared with the dry season at 960 ml/day. However, the opposite trend can be observed in urban areas, with goats producing more milk in the dry season at 650 ml/day than in the rainy season at 625 ml/day. This phenomenon can be attributed to the fact that urban residents water their animals regularly and do not often own them for the purpose of dairy farming. However, the highlands were the only regions where the differences in milk yield between the dry and rainy seasons was found to be statistically insignificant. Goats in the highlands produce an average of 1,036 ml/day in the dry season and 1,070 ml/day in the rainy season. Notably, the only parameter that exhibited a significant variance in values, particularly during the dry season, was the value for the highlands. This could be attributed to the relatively large number of respondents who answered this survey question.

Figure 7 provides additional insights into milk yield, revealing that goats in mountainous areas produce the highest amount of milk per day compared with those in urban environments or highlands and lowlands.

The reasons for this trend are not entirely clear. While the mountains of Socotra do have challenging terrain, they also have abundant grazing opportunities. Past studies have shown that access to pastures can positively impact milk yield in goats (Charpentier et al. 2019). However, the abovementioned results are confusing because goats produce less milk at higher altitudes than do goats in the lowlands. This claim was investigated by Macciotta et al. (2005), who reported that this is because it is colder at higher altitudes, and goats use more energy to thermoregulate than to produce more milk. Notably, as depicted in Fig. 7, goats produce the highest milk yields during the rainy season. Favorable weather and better mountain greenery conditions have been confirmed by Kalivodová et al. (2020), who noted that horizontal precipitation could account for up to two-thirds of the total moisture at higher altitudes. However, other factors can also influence goats’ dairy yields, such as breed, age, nutrition, environmental conditions (Erduran and Dag 2021), parity, season of kidding, and stage of lactation (Ciappesoni et al. 2004).

The analysis of milk production was conducted through a questionnaire survey; which necessitates a larger and more comprehensive study for enhancing the precision and dependability of the outcomes.

### Goat slaughter

Goats on Socotra are slaughtered in the halal way, which is no different from that of other Muslim countries.

**Fig. 8:**
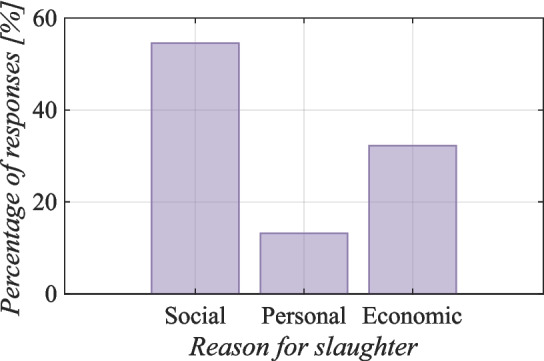
Reasons for slaughter on Socotra (Kruskal–Wallis rank sum test, value by reason Kruskal–Wallis $\chi ^2 = 171.49$, $df = 2$, $p =< 2.2 \cdot 10^{-16}$)

More than half (55%) ($p<$ 0.001) of the respondents said that they slaughtered goats mainly for social reasons, such as weddings, various celebrations, sacrifices or visitations. Furthermore, economic reasons for slaughtering, such as to sell goat meat, are the reward for their work (32%) ($p<$ 0.001). The least-represented reason for slaughtering (13%) ($p<$ 0.001) was that they slaughtered goats for their personal needs (see Fig. 8).

**Fig. 9:**
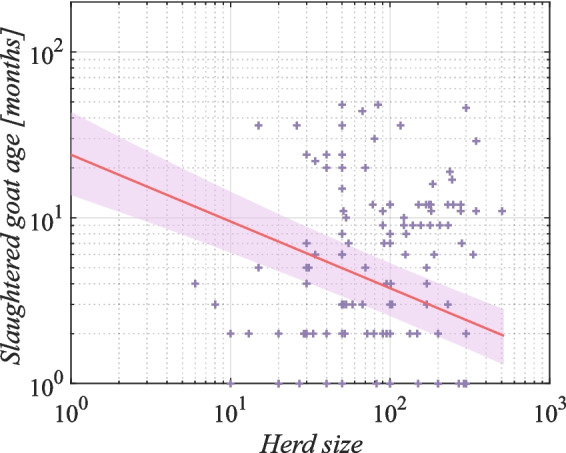
Effect of herd size on goat slaughter age on Socotra (Bayesian statistics, l-95% CI= −1.18, u-95% CI= −0.70 for number of goats)

As reported in a study by Mahanjana and Cronje (2000), goat slaughter on Socotra shares similar motivations with that in other regions. These authors reported that goats are slaughtered in connection with important family events, such as the birth of children, weddings, and the funerals of close relatives. In addition, goats are used for ceremonial purposes and to accumulate property as a form of security in cases of emergency. Similar trends were reported by Steyn (1988), who reported that people in Ciskei’s Lujiko and Nyaniso areas (South Africa) use their goats for traditional ceremonial purposes (Degen 2007) and that, as in Socotra, goats, sheep, and cattle are rarely slaughtered for domestic consumption.

Figure 9 indicates that there is a negative correlation between the number of goats owned by farmers and the age at which the goats are slaughtered. This relationship implies that the more goats farmers own, the younger the age at which the animals are slaughtered. These findings can be interpreted as an indication that a larger number of goats in a holding increases the likelihood of more frequent slaughtering and, at the same time, allows farmers to slaughter animals at a younger age.

###  Nonslaughter mortality

**Fig. 10:**
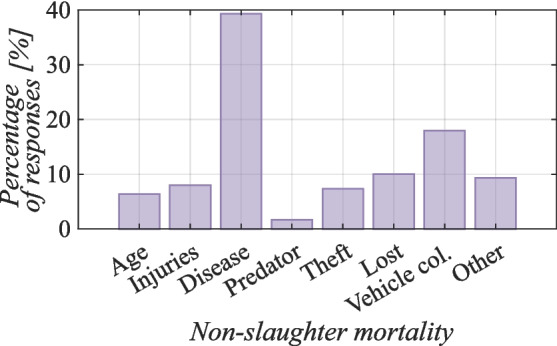
Nonslaughter mortality of goats on Socotra (Kruskal–Wallis rank sum test, value by reason Kruskal–Wallis $\chi ^2 = 232.53$, $df = 7$, $p =< 2.2 \cdot 10^{-16}$)

As shown in Fig. 10, most respondents answered that their goats died the most from disease (40% of respondents) ($p<$ 0.001), which is comparable to the results from Botswana ( southern Africa), where diseases were found to be the cause of mortality in 33% of cases (Monau et al. 2023). In Zimbabwe, only 23% of goats die of disease (Mhlanga et al. 2018). The most common diseases on Socotra, as reported by Miller and Morris (2004), include diarrhea, coughs, colds, and eye infections. Low levels of management, such as allowing goats to drink dirty river water and practicing free-range goat farming, also increase the susceptibility of these animals to disease.

Interestingly, according to the interviewees, collisions with cars were also a common cause of goat death in Socotra (18% of respondents) ($p<$ 0.001). Speed bumps on roads were built to protect goats rather than people (Scholte et al. 2007). Despite the presence of speed bumps on roads, reckless driving by local people remains a significant problem. The respondents also mentioned loss (10% of respondents) ($p<$ 0.001), theft (9% of respondents) ($p<$ 0.001), and injury (8% of respondents) ($p<$ 0.001), with goats often injured while competing for food or falling off rocks. In the last line of questioning, they state that goats on Socotra are the least likely to die naturally of old age (6% of respondents) ($p<$ 0.001) or be killed by predators (2% of respondents) ($p<$ 0.001). On the island, the main animals that eat newborn goats are the small Indian civet (*Viverricula indica*), cats (Vranik 1993) or ravens. In contrast, in Botswana ( southern Africa) (Monau et al. 2023), up to 44% of goat deaths are due to predators. Data from Zimbabwe have shown that up to 35% of goats are killed by predators there (Mhlanga et al. 2018). In our study, 9% ($p<$ 0.001) of the respondents cited other causes of death, such as mortality from a lack of water or food.

A comparison of natural (age, injuries, disease or predation) and unnatural (theft, loss, collision or other) causes of mortality revealed no significant difference (Kruskal–Wallis rank sum test, $\chi ^2 =0.249$, $df = 6$, $p = 99 \cdot 10^{-16}$).

## Conclusion

Our study investigated the socioeconomic significance of the goat population inhabiting Socotra Island. This research examined the differences in goat-rearing practices between the rainy and dry seasons and compared the data obtained from different altitudes. The survey was conducted via structured questionnaires. The findings of this study have several important implications for future practice.

One of the key findings of this study is that the Socotri people demonstrate a deeper attachment to goats than other ethnic groups in the tropical belt. They exhibit stronger social bonds with goats, with many goat keepers expressing their love for their goats as similar to their love for their children. Furthermore, they maintain herd sizes of approximately 107 goats per family on average, in contrast to the range of 10–25 goats per family in other tropical belt countries. Given the strong social bond between Socotri goat farmers and their livestock, it may be challenging to convince them to reduce the number of goats they keep. Therefore, finding alternative methods for raising goats that do not harm the environment is essential. The proposed conservation management approach must take this into account; otherwise, it may be ineffective.

According to the surveyed individuals, diseases are among the most common causes of nonslaughter mortalities in Socotra, where inadequate veterinary care threatens both the local ecosystem and the economic sustainability of the farming community. Therefore, there is an urgent need to identify and control diseases to protect human and animal health and promote the long-term ecological and economic viability of the region.

Our findings suggest that more extensive research on the socioeconomic and cultural roles of livestock is needed in Socotra. We should improve our understanding of the exact roles of the goats of Socotra in the current cultural and natural setting to completely understand their ecological impacts and population dynamics. These facets are crucial for the implementation of sustainable management strategies. Therefore, the current study emphasizes the need for further research, which may help to better manage the impacts of goats on the unique ecosystems of Socotra while respecting the importance of indigenous culture.

## Data Availability

The datasets generated and analyzed during the current study are not publicly available because the paper has not yet been published. After acceptance, it will be made available in a publicly accessible repository.

## Appendix A Collected data samples

**Table 4:** This data is mandatory. Please provide

Village	Vegetation type	Relief	Resp.
Aabab	Forest	Mountains	1
Abelhen	High shrubland	Lowland	1
Ad’ho di-Mibiriham	Grassland	Highland	1
Adihin	Grassland, High shrubland	Highland, Lowland	3
Aheft, Dzuryd	High shrubland	Lowland	1
Aheft, Rabot	High shrubland	Lowland	1
Amalih	Dwarf shrubland	Lowland	1
Aririhun	High shrubland	Lowland	1
Arishani	Dwarf shrubland	Lowland	1
Arrihun	High shrubland	Lowland	1
Ashwak	Dwarf shrubland	Lowland	1
Awdef	Grassland	Highland	1
Bahadher	Dwarf shrubland	Lowland	1
Bed Lehayhen	High shrubland	Highland	2
Beytuh	Dwarf shrubland	Lowland	1
Bidholeh	Dwarf shrubland	Lowland	1
Bizidig	Dwarf shrubland	Lowland	2
Buoded	High shrubland	Highland	1
Deqamenh	High shrubland	Lowland	1
Di Hahus	Mountain mosaic of grass	Mountains	1
Di hamry	High shrubland	Lowland	2
Di-farq	Mountain mosaic of grass	Mountains	2
Di-filaq	Grassland	Highland	1
Di-hamero	Grassland	Highland	1
Di-hashus	Mountain mosaic of grass, Grassland	Mountains, Highland	3
Di-hof	High shrubland	Highland	2
Di-Noban	High shrubland	Lowland	2
Di-Seberho	Dwarf shrubland	Lowland	1
Di-Selmeho	High shrubland	Lowland	1
Dibuderiahen	High shrubland, Woodland	Highland, Lowland	2
Diham	High shrubland	Lowland	2
Diherso	High shrubland	Lowland	1
Dil-Ober	Grassland	Highland	2
Dilmakehur	Shrubland	Mountains	1
Dimakehr	High shrubland	Highland	2
Dimakehur	Shrubland	Mountains	1
Dithak	Shrubland	Mountains	1
Eserhe	Grassland	Highland	1
Eyroh	High shrubland	Highland	1
Felginitin	High shrubland	Highland	1
Figiri	High shrubland	Highland	1
Gehended	Dwarf shrubland	Lowland	1
Genoti	Dwarf shrubland	Lowland	2
Haderhen	High shrubland	Highland	1
Hadibo	Dwarf shrubland, City	Lowland, City	7
Hamiro	High shrubland	Highland, Lowland	3
Hazahaz	Dwarf shrubland	Lowland	1
Hilho	Grassland	Highland	1
Imtey	Dwarf shrubland	Lowland	1
Kabhaitin	Woodland	Lowland	1
Kadudihan	High shrubland	Lowland	2
Khofgi	High shrubland	Highland	1
Kusaf	High shrubland, Grassland	Highland	2
Lamuh	Grassland	Highland	2
Lisko	High shrubland	Lowland	2
Liyheh	Shrubland	Mountains	1
Lyhub	High shrubland	Highland	1
Lyieh	High shrubland	Highland	1
Ma’anefo	High shrubland	Lowland	1
Mahfer	Grassland, High shrubland	Highland	2
Masaaba	Grassland	Highland	1
Medoqoh	High shrubland, Mountain mosaic of grass	Highland, Mountains	4
Meharef	Dwarf shrubland	Lowland	1
Menhi	Grassland, Shrubland	Highland, Mountains	3
Mokhar	Dwarf shrubland	Lowland	1
Mouri	Dwarf shrubland, Grassland	Lowland, Highland	2
Nuwtat	High shrubland	Highland	1
Pataria	Dwarf shrubland	Lowland	1
Qabhitin	High shrubland	Lowland	2
Qalansiyah	Dwarf shrubland, Woodland, Grassland, High shrubland	Lowland	5
Qaraad	Mountain mosaic of grass	Highland	1
Qaris	Grassland	Highland	2
Qataninhin	High shrubland	Highland	1
Qermeh	Dwarf shrubland	Lowland	1
Qeshebeb	Dwarf shrubland	Lowland	3
Qowshen	High shrubland	Highland	1
Quariah	High shrubland	Lowland	1
Quashur	Mountain mosaic of grass	Mountains	1
Reged	Grassland	Highland	2
Rehen	Dwarf shrubland	Lowland	1
Reqabunh	High shrubland	Highland	1
Reqfef	Forest, Shrubland	Mountains	2
Rizehum	Forest	Mountains	1
Rokeb	Grassland	Highland	1
Rokhan	Dwarf shrubland	Lowland	1
Sanq	Grassland	Highland	1
Shezarihen	High shrubland	Lowland	1
Shibon	Highland grassland	Highland	2
Sirhen	Grassland	Highland	1
Stero	Dwarf shrubland	Lowland	2
Terebek	Dwarf shrubland,High shrubland	Lowland	8
Toys	Grassland	Highland	1
Truba	Grassland,Shrubland,High shrubland	Highland, Mountains,Lowland	5
Tuhe	Dwarf shrubland	Lowland	1
Ziho	High shrubland	Highland	1
Zuhu	High shrubland	Highland	1

Bold values (No Change)
